# La odontología del futuro: un enfoque integrado en salud familiar y comunitaria

**DOI:** 10.21142/2523-2754-1103-2023-160

**Published:** 2023-09-26

**Authors:** Alfredo Cueto U

**Affiliations:** Universidad de Valparaíso, Facultad de Odontología, Valparaiso, Chile. alfredocuetourbina@yahoo.es Universidad de Valparaíso Universidad de Valparaíso Facultad de Odontología Valparaiso Chile alfredocuetourbina@yahoo.es

En un mundo en constante evolución, la odontología se enfrenta a nuevos retos y oportunidades que nos llevan a repensar nuestro enfoque tradicional. Históricamente, las universidades han desempeñado un papel crucial en la formación de profesionales de la salud bucodental, enfocándose en la docencia, investigación y vinculación con el entorno. Sin embargo, con la llegada de la odontología digital y la inteligencia artificial, vislumbramos un panorama donde ciertas actividades odontológicas podrían automatizarse, transformando la forma en que abordamos la salud oral.

La automatización de ciertos procedimientos, inicialmente al alcance de unos pocos privilegiados, podría extenderse masivamente en la población a medida que los costos disminuyan y las tecnologías sean más accesibles. Esta revolución tecnológica promete mejorar la eficiencia y precisión en la atención odontológica, pero no podemos perder de vista el verdadero propósito de nuestra disciplina: proporcional salud y, a la vez, atender las necesidades de salud bucodental de la población para fomentar la calidad de vida de manera integral.

Es cierto que, hasta ahora, gran parte de nuestra práctica ha estado centrada en la rehabilitación dental, con un enfoque reactivo en el tratamiento de enfermedades bucodentales. Sin embargo, esta aproximación se muestra insuficiente para abarcar las necesidades de toda la población. Problemas de cobertura, falta de recursos, gestión inadecuada de programas y barreras de acceso dificultan nuestro alcance y contribuyen a mantener un alto nivel de deterioro de la salud bucodental, especialmente en países de Latinoamérica.

Es momento de repensar nuestra perspectiva y abrazar un enfoque más amplio y centrado en la prevención y promoción de la salud oral. La Asociación Dental Internacional (FDI) destaca que muchas enfermedades bucodentales comparten factores de riesgo con otras enfermedades no transmisibles, como enfermedades cardiovasculares, diabetes y respiratorias ^1^. Esta interconexión entre la salud bucodental y la salud general nos ofrece una sólida base para una integración más estrecha de ambas áreas.

Para lograr una transformación significativa, deberíamos enfocarnos en la salud familiar y comunitaria. El núcleo familiar es un recurso vital para la promoción de la salud y prevención de enfermedades. Al adoptar un enfoque de salud familiar, no solo impactamos positivamente al individuo, sino a todos los miembros de la familia, creando hábitos saludables que perdurarán en el tiempo.

Asimismo, es esencial incorporar un enfoque de salud comunitaria, involucrando activamente a la comunidad en la planificación, administración, gestión y control de las acciones de salud ^2^. Una comunidad comprometida y participativa podrá contribuir con su conocimiento, experiencia para abordar problemas y encontrar soluciones, generando un impacto significativo y sostenible en la salud bucodental de sus integrantes ^3^, trabajado desde la atención primaria en salud en especial para los más vulnerables, para disminuir las inequidades en salud.

En esta travesía hacia una odontología del futuro, las universidades deberían asumir un rol fundamental. Los profesionales y los que están en la docencia deberían apostar por una formación transversal y transdisciplinaria, promoviendo la integración de la salud oral con la salud general y adoptando una mirada holística de los pacientes valorando sus contextos familiares y comunitarios.

La tecnología seguirá avanzando y es nuestro deber aprovechar su potencial para mejorar la calidad de vida de las personas. No obstante, nunca debemos olvidar que nuestro objetivo va más allá de las restauraciones dentales y sus períodos de reemplazo, que tienen poco impacto en la población ^4^. Debemos trabajar incansablemente para fomentar la salud bucodental en la población, prevenir enfermedades y promover una vida activa y saludable.

En el camino hacia una odontología del futuro, el compromiso con nuestra comunidad y la visión de una salud integral deben guiar cada paso que damos como profesionales. Juntos, como universidad, como profesionales y como comunidad, influyendo en la toma de decisiones a nivel comunal y nacional, podremos alcanzar el anhelado objetivo de mejorar la salud bucodental en Latinoamérica y, más allá, lo que generará un impacto positivo y duradero en la vida de las personas que servimos.
